# Activation of myeloid dendritic cells, effector cells and regulatory T cells in lichen planus

**DOI:** 10.1186/s12967-016-0938-1

**Published:** 2016-06-10

**Authors:** Rosana Domingues, Gabriel Costa de Carvalho, Valéria Aoki, Alberto José da Silva Duarte, Maria Notomi Sato

**Affiliations:** Laboratory of Dermatology and Immunodeficiencies, LIM-56, Department of Dermatology, Medical School, University of São Paulo, Institut of Tropical Medicine of São Paulo, Av. Dr. Enéas de Carvalho Aguiar, 500, 3rd floor 24, São Paulo, 05403-000 Brazil; Dermatological Outpatient Clinic, Hospital das Clínicas, Medical School of the University of São Paulo, São Paulo, Brazil

**Keywords:** Lichen planus, Toll-like receptor, Dendritic cells, Regulatory T cells, Polyfunctional T cells

## Abstract

**Background:**

Lichen planus (LP) is a chronic mucocutaneous inflammatory disease. Evaluating the balance between regulatory T cells and effector T cells could be useful for monitoring the proinflammatory profile of LP. Therefore, this study aimed to assess populations of dendritic cells (DCs) and regulatory and effector T cells in peripheral blood samples collected from patients with LP to evaluate the polyfunctionality of T cells upon toll-like receptor (TLR) activation.

**Methods:**

Peripheral blood mononuclear cells collected from 18 patients with LP and 22 healthy control subjects were stimulated with agonists of TLR4, TLR7, TLR7/TLR8 or TLR9. Frequencies of circulating IFN-α^+^ plasmacytoid DCs (pDCs); TNF-α^+^ myeloid DCs (mDCs); regulatory T cells (Tregs); and IL-17-, IL-10-, IL-22-, TNF-, and IFN-γ-secreting T cells were assessed via flow cytometry.

**Results:**

The frequencies of regulatory CD4^+^ and CD8^+^CD25^+^Foxp3^+^CD127^low/−^ T cells and TNF-α^+^ mDCs were induced following activation with TLR4, TLR7 and TLR8 agonists in the LP group. Moreover, increased baseline frequencies of CD4^+^IL-10^+^ T cells and CD8^+^IL-22^+^ or IFN-γ^+^T cells were found. In the LP group, TLR4 activation induced an increased frequency of CD4^+^IFN-γ^+^ T cells, while TLR7/8 and staphylococcal enterotoxin B (SEB) activation induced an increased frequency of CD8^+^ IL-22^+^ T cells. An increased frequency of polyfunctional CD4^+^ T cells that simultaneously secreted 3 of the evaluated cytokines (not including IL-10) was verified upon TLR7/8/9 activation, while polyfunctional CD8^+^ T cells were already detectable at baseline.

**Conclusions:**

TLR-mediated activation of the innate immune response induced the production of proinflammatory mDCs, Tregs and polyfunctional T cells in patients with LP. Therefore, TLR activation has an adjuvant role in inducing both innate and adaptive immune responses.

**Electronic supplementary material:**

The online version of this article (doi:10.1186/s12967-016-0938-1) contains supplementary material, which is available to authorized users.

## Background

Lichen planus (LP) is a chronic inflammatory disease that affects skin and mucous membranes and can be mediated by T cells. Although the aetiology is unknown, LP has been associated with human hepatitis C [1] and human herpes virus type 7 infections [2].

Previously, we verified that patients with LP exhibit dysfunctional cytokine secretion by peripheral blood mononuclear cells (PBMCs) after Toll-like receptor (TLR) activation. This phenotype was mainly related to the activation of intracellular TLRs, including Poly-RIG/TLR3, imiquimod/TLR7, CL097/TLR7/8, and CpG/TLR9; similar events occur during viral infection [3]. Moreover, we showed that up-regulated expression of factors related to the type I IFN axis and antiviral restriction factors as well as enhanced expression of human endogenous retroviruses are characteristic of skin lesions in patients with cutaneous LP, attributing a viral aetiology to LP pathogenesis [4].

Plasmacytoid dendritic cells (pDCs) are present in large numbers in LP skin lesions and have the ability to produce large amounts of IFN-α in response to TLR7 and TLR9 stimulation, showing their key role in antiviral responses [2]. In contrast, myeloid dendritic cells (mDCs) with immunoregulatory characteristics are also found in LP lesions [5]. Understanding the cytokine secretion profiles of pDCs and mDCs in peripheral blood could provide insights into how dendritic cells (DCs) impact T cell functions and the balance that exists between regulatory and effector responses in LP.

Regulatory T cells (Tregs) play a crucial role in immune tolerance and the prevention of autoimmune and inflammatory diseases [6, 7]. Tregs express the transcription factor Foxp3 and are classified according to their origin as either thymus-derived Tregs (tTreg), which recognize self antigens, or peripherally derived Tregs (pTreg), which recognize antigens from microbiota and allergens as well as alloantigens [8]. In oral and cutaneous LP lesions, Foxp3^+^ T cells infiltrate both the epidermis and dermis. Moreover, these cells are also found at an increased frequency in the peripheral blood of patients with oral LP (OLP) [9, 10]. In cutaneous LP, the presence of these cells in peripheral blood has not been evaluated to date.

Increased frequencies of CD4^+^IFN-γ^+^ T cells and CD4^+^IFNγ^+^IL-17^+^ T cells have been found in the peripheral blood of patients with OLP after stimulation with phorbol myristate acetate (PMA) and ionomycin [11]. Furthermore, CD8^+^ T cells play an important role in LP skin lesions by destroying keratinocytes via the induction of apoptosis through FasL expression, the activation of granzymes and perforin, and the secretion of TNF-α [12]. A link between human papillomavirus virus infection and OLP pathogenesis has been described, including the identification of a massive clonal expansion of CD8^+^ T cells with an increased frequency of HPV-16-specific CD8^+^ T cell subpopulations in patients with OLP [13]. However, in LP, it is unknown whether TLR-mediated activation of cells in peripheral blood may contribute to the adaptive responses of CD4^+^ or CD8^+^ T cells, which are mainly related to the secretion of IL-17 (Th17/Tc17 cells), IL-22 (Th22/Tc22 cells) and IFN-γ (Th1/Tc1 cells).

To date, the generation of polyfunctional responses in skin diseases has only been studied in psoriasis and atopic dermatitis [14–16]. Polyfunctionality is defined as the ability to simultaneously produce multiple cytokines and has an important role in viral control. In HIV and tuberculosis, polyfunctionality has been associated with better vaccine response and slower progression to disease. Furthermore, polyfunctional cells are targeted when designing vaccines and immunotherapies that are mediated though cellular responses [17–19].

In this work, we demonstrated that patients with LP have increased frequencies of TNF-α^+^ mDCs and CD4^+^/CD8^+^ Tregs in their peripheral blood. We also verified that TLR activation led to impaired IL-10 production by CD4^+^ T cells and the presence of Th22/Tc22 cells in peripheral blood. TLR-mediated signalling events induce DC maturation and are crucial to inducing monofunctional or polyfunctional responses by CD4^+^ and CD8^+^ T cells in patients with LP. Therefore, TLR activation could be useful for the immunomodulation of LP.

## Methods

### Study population

The current study enrolled patients with cutaneous LP (n = 18; 3 males, 15 females) from the Dermatological Outpatient Clinic of the Hospital das Clínicas de São Paulo (HC-FMUSP) as well as healthy individuals as controls (n = 22; 5 males, 17 females). The majority cohort of patients with LP had the cutaneous form of LP and association with oral form was observed in 3 patients. Patients with other forms of LP, such as lichen planopilaris or drug-induced LP, as well as patients with LP associated with autoimmune diseases and other skin diseases were not included. The majority of the patients had at least two affected limbs, with papules involving up to 30 % of the trunk. None of the patients took any type of topical or systemic corticosteroids, retinoids or immunosuppressants for 1 month prior to blood collection. A serological screening for hepatitis C was performed. The median age was 43.9 ± 13.5 years (range: 22–65 years) for the patients with LP and 40.5 ± 14.3 years (range: 20–71 years) for the healthy individuals. All the subjects provided written informed consent under the approval of the São Paulo University Institutional Use Committee (CAPPesq no. 0709/11).

### Flow cytometry for Treg analysis

To analyse Treg populations, PBMCs were isolated from heparinized venous blood by Ficoll-Hypaque gradient centrifugation (GE Healthcare Bio-Sciences AB, Uppsala, Sweden) and diluted in RPMI medium supplemented with 10 % AB human serum (Sigma, St. Louis, MO, USA). The PBMCs were incubated with antibodies against the following proteins at 4 °C for 30 min: CD3 (S4.1, Qdot, Invitrogen, Carlsbad, CA, USA), CD4 (RPA T4, Horizon V500), CD8 (RPA T8, Alexa Fluor 700), CD45RA (HI 100, APC-H7), CCR7 (3D12, Alexa Fluor 647), and CD127 (HIL-7R-M21, RPE-Cy7). All antibodies were from BD Biosciences (San Jose, CA, USA). The cells were then fixed, permeabilized and incubated with an anti-Foxp3 antibody (PCH101, e-Bioscience, San Diego, CA, USA) at 4 °C for 30 min. The Tregs were characterized as CD3^+^CD4^+^CD25^+^CD127^low/−^Foxp3^+^ or CD8^+^CD25^+^CD127^low/−^Foxp3^+^ and as thymus-derived Tregs (tTregs; CD45RA^+^Foxp3^low^) or peripherally derived Tregs (pTregs; CD45RA^−^Foxp3^high^). A total of 400,000 events were acquired using a flow cytometer (LSR Fortessa, BD Biosciences, USA) with FACS-Diva software (BD Bioscience). The data were analysed using FlowJo software, version 9.4.11 (Tree Star, Inc., Ashland, OR, USA).

### Evaluation of DCs and effector T cells

Cultures of PBMCs (2.0 × 10^6^ cells/500 µL) were incubated in 48-well plates (Costar, Cambridge, MA, USA) in RPMI 1640 medium with ligands for TLR4 (lipopolysaccharide, 2 µg/mL), TLR7 (imiquimod, 2.5 µg/mL), TLR7/TLR8 (CL097, 5 µg/mL), or TLR9 (oligodeoxynucleotide CpG, 4 µM/mL) for 16 h or SEB (enterotoxin B of *Staphylococcus aureus*, 1 μg/mL) as a positive control for 6 h at 37 °C under 5 % CO_2_. The stimulus concentrations and culture times were previously determined using a dose–response curve (Cardoso EC, 2013). All the ligands were obtained from Invivogen (San Diego, CA, USA). Brefeldin A (10 µg/mL, Sigma) was added to the cells 4 h after the beginning of the culture. mDCs were characterized as Lin^−^CD11c^+^HLA-DR^+^, and pDCs were characterized as Lin^−^CD123^+^HLA-DR^+^. After the Brefeldin A incubation, the cells were washed and incubated with human IgG for 10 min followed by staining with LIVE/DEAD (Invitrogen), fixation with Cytofix/Cytoperm solution (BD Biosciences) for 20 min and a wash in Perm/Wash solution. The cells were stained with antibodies against the following proteins: Lineage 1 cocktail (SK-7, Lin1, a mix of CD3, CD14, CD16, CD19, CD20 and CD56) (FITC), HLA-DR (G46-6, Horizon V500), CD123 (7G3, PERCP Cy5.5), CD11c (B-ly6, Alexa fluor 700), IFN-α (7N4-1, Horizon V500) and TNF-α (6401.111, PE). For monofunctional and polyfunctional T cell analyses, PBMCs were stained with LIVE/DEAD (Invitrogen, viability marker), fixed and permeabilized with Cytofix/Cytoperm solution (BD Biosciences) and then stained with antibodies against CD3 (SP34-2, BV605), CD4 (RPA-34, Horizon V500), CD8 (RPA-T8, PERCP Cy5.5), TNF (MAb11, PECy7), IL-10 (JES3-19F1, APC), CD38 (HIT2, Alexa Fluor 700) and IFN-γ (B27, Horizon V450) from BD Biosciences and antibodies against IL-22 (22URTI, PE) and IL-17 (N49-653, Alexa Fluor 488) from eBioscience. A total of 500,000 events were acquired with a flow cytometer (LSRFortessa, BD) and analysed with FlowJo software. The fluorescence minus one (FMO) protocol was used for all analyses to determine the gates. Boolean gate arrays were then created using FlowJo software. This analysis determined the frequency of each cytokine based upon all possible combinations of cytokines. To analyse the polychromatic flow cytometry data and to generate graphical representations of the T cells, the SPICE Program was used (Version 2.9, Vaccine Research Center, NIAID, NIH).

### Statistical analysis

The Mann–Whitney U test was used to compare variables between the patients with LP and the healthy controls. P ≤ 0.05 was considered significant.

## Results

### mDCs are responsive to TLR activation in LP

Previously, we verified that mononuclear cells from patients with LP have altered cytokine secretion upon activation of TLRs with synthetic ligands. This finding mainly applies to intracellular TLR7/TLR8 and TLR9 signalling [3]. One cytokine that is abundantly produced in PBMCs upon TLR activation is TNF [3]. Based on these studies, we analysed the frequency of TNF-α^+^ mDCs within populations of PBMCs [20]. To evaluate the responsiveness of DCs (mDCs and pDCs), populations of PBMCs were assessed using flow cytometry to determine the frequencies of TNF-α^+^ mDCs and IFN-α^+^ pDCs after stimulation with ligands for TLR4 (LPS), TLR7 (imiquimod) and TLR7/8 (CL097), and TLR9 (CpG) or with SEB as a positive control. The gating strategy used to evaluate the pDCs and mDCs is depicted in Additional file 1: Figure S1.

**Fig. 1:**
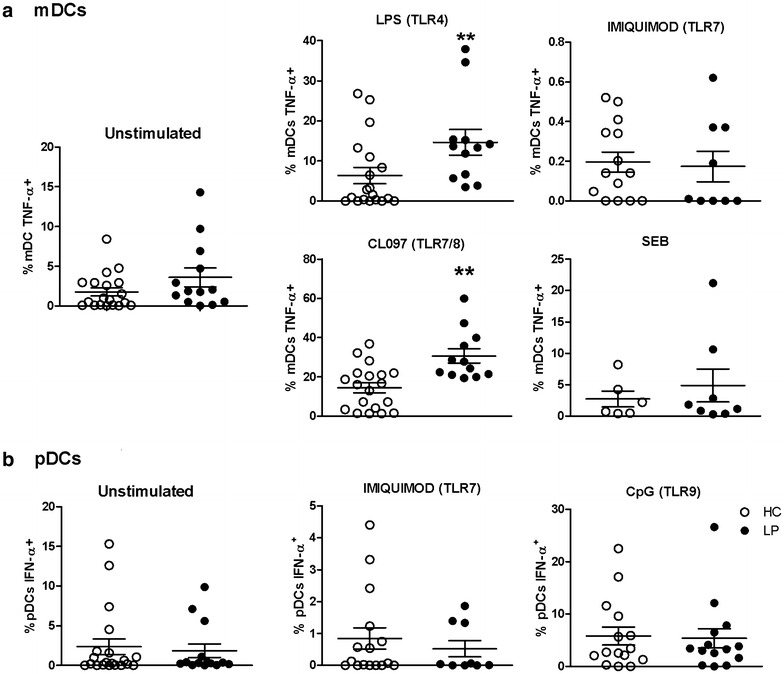
TLR activation increased the frequency of TNF-α^+^ mDC populations in patients with LP. PMBCs from patients with LP (n = 13, *closed circles*) and HC (n = 19, *open circles*) were left unstimulated or were stimulated with the following TLR agonists: LPS/TLR4, imiquimod/TLR7, CL097/TLR7-8, CpG/TLR9 and SEB. The cells were incubated with the agonists for 16 h and assessed using flow cytometry. **a** Production of TNF-α in mDCs (CD11c^+^HLA-DR^+^TNF-α^+^). **b** IFN-α secretion by pDCs (CD123^+^HLA-DR^+^IFN-α^+^) was subtracted from unstimulated levels. The results are shown as the mean ± SEM. *P < 0.01 when compared with the HC group

As shown in Fig. 1, an increased frequency of CD11c^+^HLA-DR^+^TNF-α^+^ mDCs was found in the LP samples after stimulation with LPS/TLR4 and CL097/TLR7-TLR8. No difference was observed following TLR7 activation, indicating that activation of TLR8 using the CL097 compound in mDCs potently induced TNF-α secretion. To induce type I interferon secretion by CD123^+^HLA-DR^+^ pDCs, we used TLR7 and TLR9 agonists, which resulted in similar frequencies of IFN-α^+^ pDCs in the LP and HC groups (Fig. 1b).

### Patients with LP have an increased frequency of Tregs in peripheral blood

To verify that T cells were present in the patients with LP, we evaluated populations of tTregs and pTregs, derived from thymic or peripheral T cells, respectively [8]. To accomplish this goal, we evaluated the frequencies of CD4^+^ and CD8^+^Foxp3^+^ Tregs in peripheral blood samples collected from the LP and HC groups. The gating strategy used to identify Tregs is shown in Additional file 2: Figure S2.

**Fig. 2:**
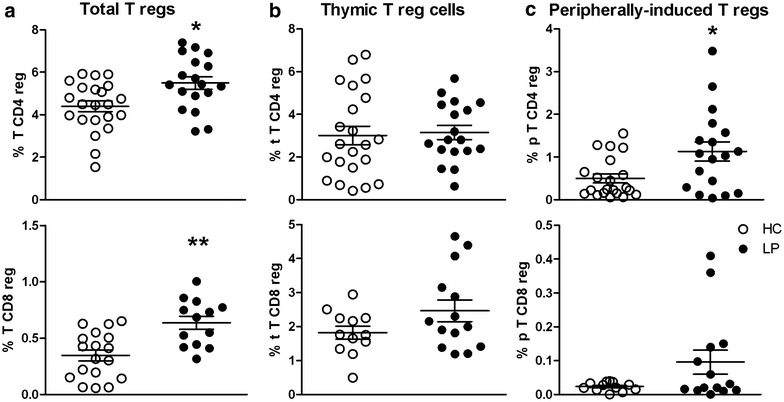
The frequencies of CD4^+^ and CD8^+^ Treg populations were increased in the peripheral blood of patients with LP. PBMCs obtained from patients with LP (n = 18, *closed circles*) and HC (n = 22, *open circles*) were assessed by flow cytometry for the presence of (**a**) CD4^+^ Tregs (CD3^+^CD4^+^CD25^+^Foxp3^+^CD127^low/−^) and CD8^+^ Tregs (CD3^+^CD8^+^CD25^+^Foxp3^+^CD127^low/−^), **b** thymus-derived CD4^+^ Tregs (CD4^+^CD45RA^+^Foxp3^low^) and thymus-derived CD8^+^ Tregs (CD8^+^CD45RA^+^Foxp3^low^), and (**c**) peripherally derived CD4^+^ Tregs (CD4^+^CD45RA^−^Foxp3^high^) and peripherally derived CD8^+^ Tregs (CD8^+^CD45RA^−^FoxP3^high^). The results are shown as the mean ± SEM. *P < 0.05, **P < 0.01 when compared with the HC group

The patients with LP exhibited increased frequencies of CD4^+^CD25^+^Foxp3^+^CD127^low/−^ T cells and CD8^+^ Tregs (Fig. 2a). Among the different subtypes of Tregs analysed, we verified that similar frequencies of CD4^+^ and CD8^+^ tTregs were present in both groups (Fig. 2b), while the patients with LP had a higher frequency of CD4^+^ pTregs than the healthy controls (Fig. 2c).

### Altered cytokine responses in CD4^+^ and CD8^+^ T cells were induced by TLR activation in patients with LP

Previously, we verified that TLR activation produces an adjuvant effect by inducing cytokine production in PBMCs [3]. In the current study, we evaluated how stimulation of the innate immune system through TLR activation in T cells influences LP. Additionally, we stimulated PBMCs with TLR agonists such as LPS/TLR4, CL097/TLR7-8, CpG/TLR9 and SEB and then assessed intracellular levels of IFN-γ, IL-10, IL-22 and TNF in CD4^+^ and CD8^+^ T cells. The gating strategy used to quantify cytokines secreted from T cells is depicted in Additional file 3: Figure S3 (a, b).

**Fig. 3:**
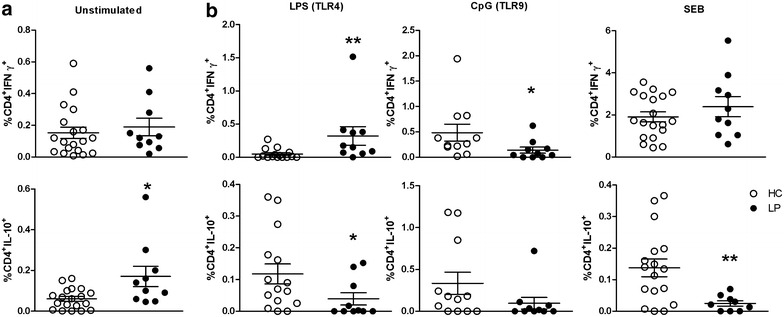
Altered cytokine secretion by CD4^+^ T cells induced by TLR activation in patients with LP. PBMCs from patients with LP (n = 13, *closed circles*) and HCs (n = 19, *open circles*) were left unstimulated (**a**) or were stimulated with the TLR agonists LPS/TLR4, CpG/TLR9 and SEB for 16 h (**b**) and then assessed by flow cytometry for IFN-γ and IL-10 secretion. The frequencies of stimulated CD4^+^ T cell populations were subtracted from those of unstimulated populations. The results are shown as the mean ± SEM. *P < 0.05, **P < 0.01 when compared with the HC group

We assessed the frequencies of CD4^+^ T cells that secreted IFN-γ and IL-10 (Fig. 3) and CD8^+^ T cells that secreted IFN-γ, IL-22 and TNF (Additional file 4: Figure S4) because these cytokines are specifically modified after TLR activation. Relative to the HC group, in the patients with LP, CD4^+^IFN-γ^+^ T cell frequency increased after stimulation with a TLR4 agonist and decreased after TLR9 activation, but it was not altered by SEB stimulation (Fig. 3a). Moreover, the patients with LP had a higher frequency of unstimulated CD4^+^IL-10^+^ T cells, whereas this population decreased following TLR4 or SEB stimulation.

Unstimulated CD8^+^ T cells exhibited increased production of proinflammatory cytokines, such as IFN-γ and IL-22 (Additional file 4: Figure S4). After stimulation with CL097/TLR7-8 or SEB, the LP group displayed a high frequency of IL-22^+^ cells (Additional file 4: Figure S4b). Upon TLR9 stimulation, the frequency of CD8^+^ T cells secreting TNF or IFN-γ (Additional file 4: Figure S4c) decreased in the LP group.

We also evaluated the ratios between Th17 cells and Tregs and Th1 cells and Tregs using baseline data (Additional file 5: Figure S5). These ratios were similar between the LP and HC groups.

### Patients with LP exhibit increased frequencies of Th22 and Tc22 cells

**Fig. 4:**
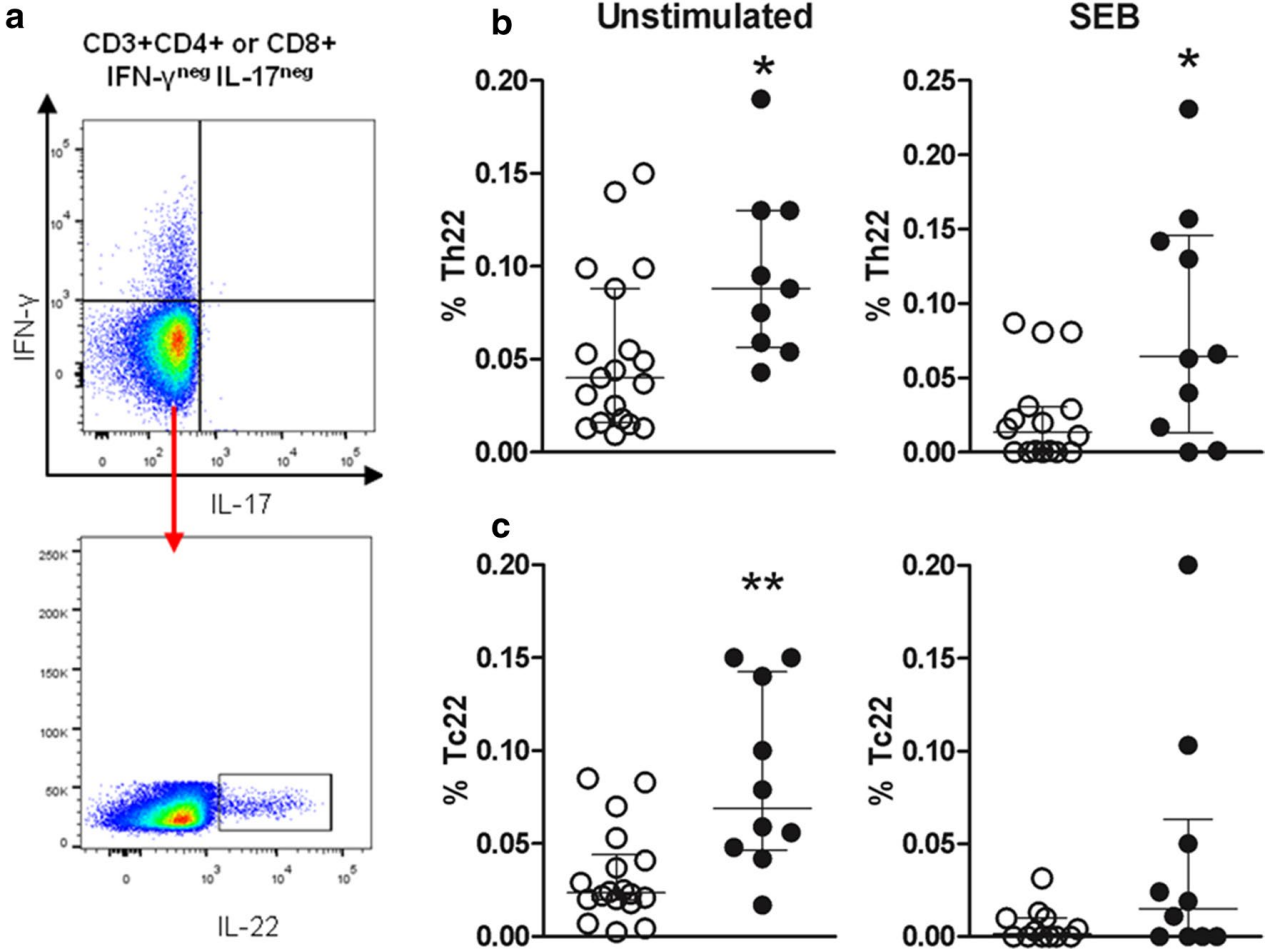
Frequencies of Th22 and Tc22 cells in HCs and patients with LP. PBMCs obtained from patients with LP (n = 10, *black circles*) and HCs (n = 19, *white circles*) were left unstimulated or were stimulated with SEB for 16 h and assessed by flow cytometry. A representative gating strategy for the selection of CD4^+^ T cells is shown; the same strategy was used to select CD8^+^ T cells (**a**). Production of CD3^+^CD4^+^ and CD3^+^CD8^+^ (IFN-γ^−^IL-17^−^) T cells at basal levels (**b**) and following stimulation with SEB (subtracted from baseline levels) (**c**). The results are shown as the mean ± SEM. *P < 0.05, **P < 0.01 when compared with the HC group

To evaluate the frequencies of Th22 and Tc22 cells in peripheral blood, we analysed production of the cytokines IL-17, IFN-γ and IL-22 in T cells via flow cytometry. Th22 and Tc22 cells are IL-17^−^IFN-γ^−^IL22^+^, and the gating strategy used to identify these cells is shown in Fig. 4a. Under no-stimulation conditions, the LP group exhibited increased frequencies of Th22 and Tc22 cells compared to the HC group; this trend remained for Th22 cells following stimulation with SEB (Fig. 4b).

### TLR activation induces the production of polyfunctional T cells in patients with LP

**Fig. 5:**
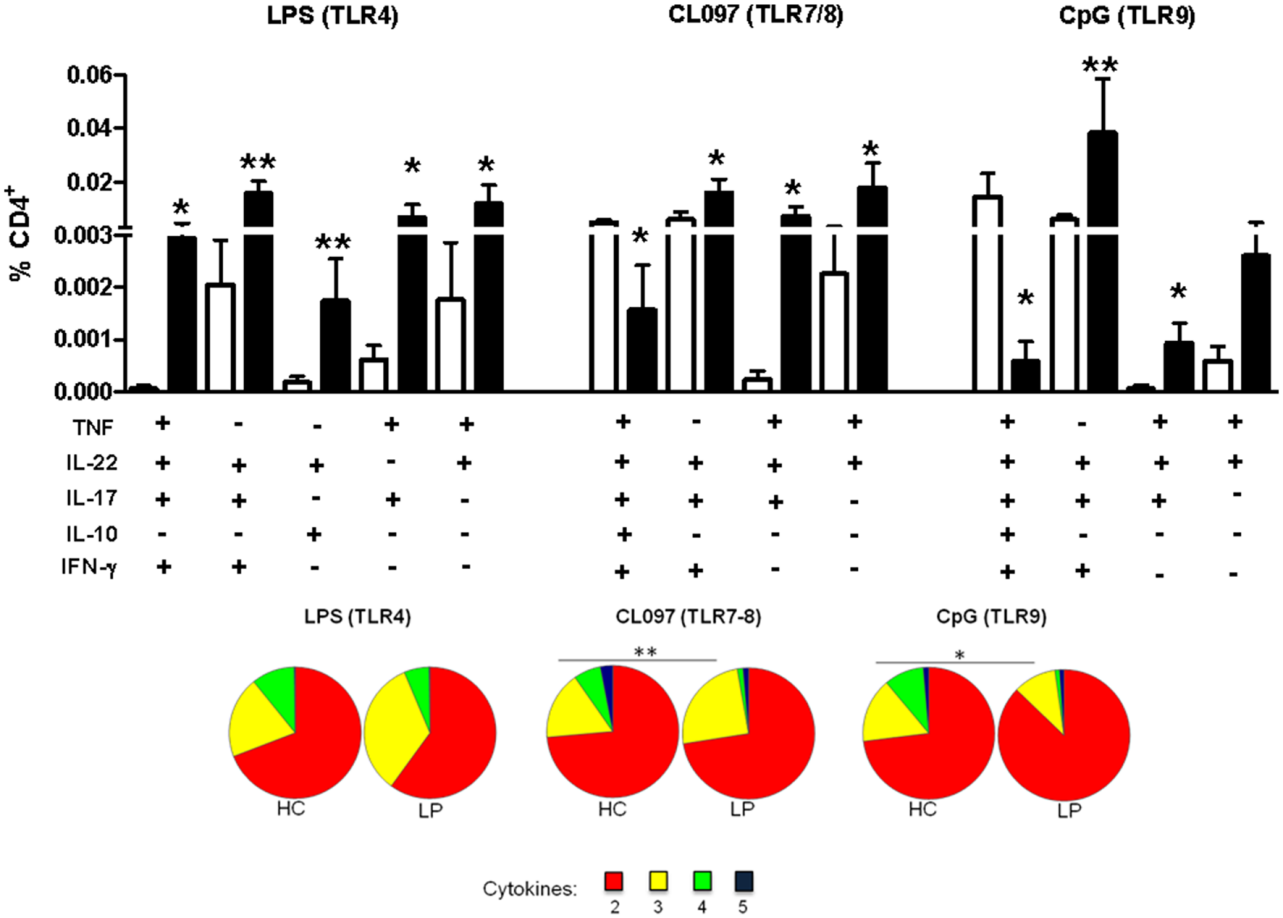
Polyfunctional CD4^+^ T cells are induced upon TLR activation in patients with LP. PBMCs obtained from patients with LP (n = 10, *black bars*) and HCs (n = 19, *white bars*) were left unstimulated or were stimulated with the following TLR agonists: LPS/TLR4, CL097/TLR7-8, CpG/TLR9 and SEB. The cells were incubated with the agonists for 16 h and assessed using flow cytometry. Cytokine production (TNF, IL-22, IL-17, IL-10 and IFN-γ) from stimulated CD3^+^CD4^+^ T cells was subtracted from the baseline levels. The results are shown as the mean ± SEM. *P < 0.05, **P < 0.01 when compared with the HC group

Next, we evaluated whether stimulation of PBMCs with TLRs could induce the production of polyfunctional T cells in patients with LP. To accomplish this, we assessed levels of the cytokines IL-17, IL-10, IFN-γ, TNF and IL-22 in T cells via flow cytometry using a Boolean gating strategy, which is described in Additional file 3: Figure S3c. As shown in Fig. 5, the patients with LP had fewer CD4^+^ T cells that simultaneously secreted the 5 evaluated cytokines following treatment with CL097 and CpG. However, TLR4 or TLR7/8 and TLR9 activation induced the production of some of these cytokines in T cells from patients with LP, although these cells did not appear to produce IL-10. Moreover, in the patients with LP, we observed an increased frequency of CD4^+^ T cells simultaneously secreting 3 of the evaluated cytokines compared to the HC following treatment with CL097 or CpG (pie chart, yellow slice). Notably, the most prominent combinations of 3 cytokines in this context included IL-22, IL-17, IFN-γ, or TNF, but not IL-10.

**Fig. 6:**
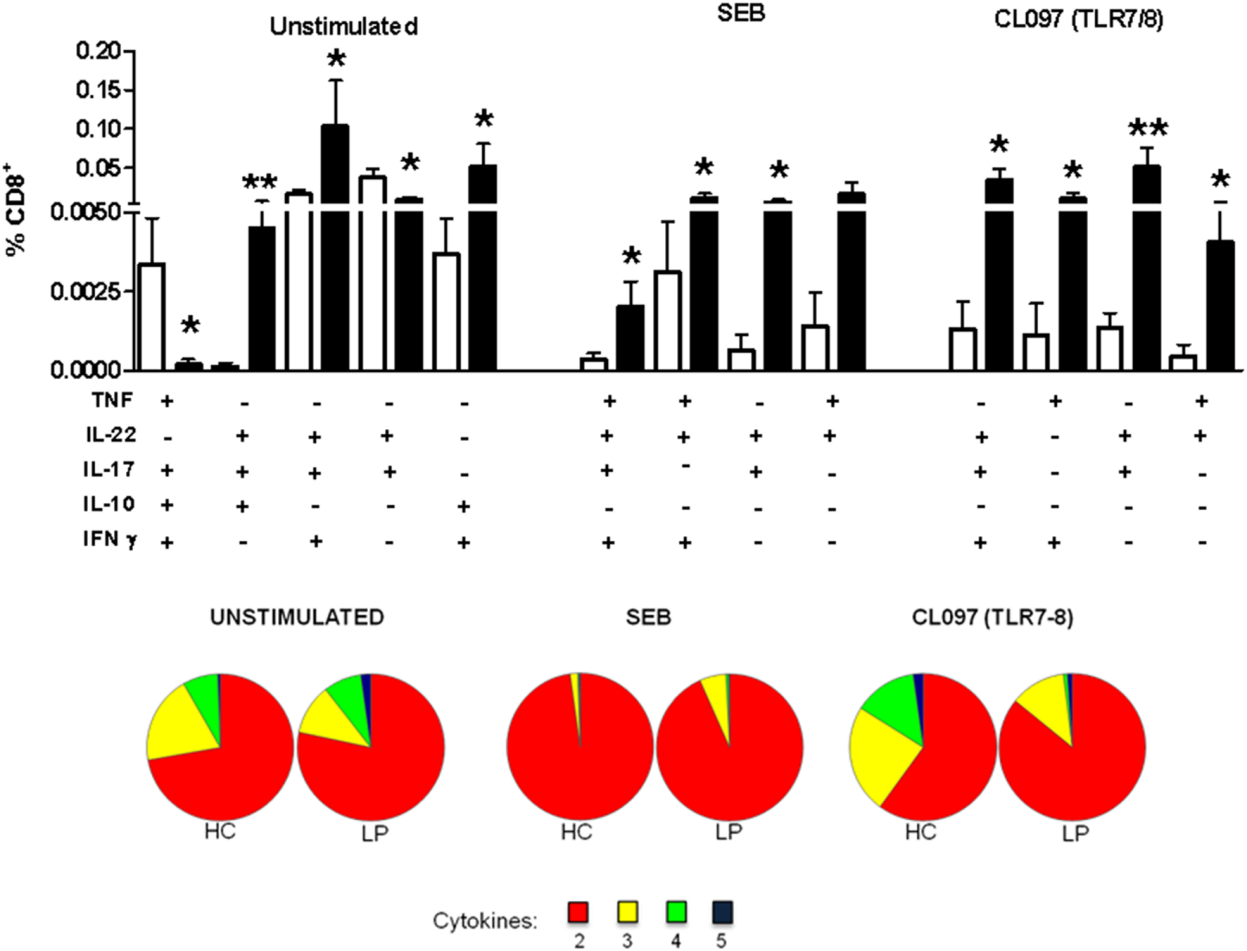
Polyfunctional CD8^+^ T cells are induced upon TLR activation in patients with LP. PBMCs obtained from patients with LP (n = 10, *black bars*) and HCs (n = 19, *white bars*) were left unstimulated or were stimulated with the TLR agonists CL097/TLR7-8 and SEB for 16 h and assessed by flow cytometry. Cytokine production (TNF, IL-22, IL-17, IL-10 and IFN-γ) by stimulated CD3^+^CD8^+^ T cells was subtracted from the baseline levels. The *pie chart* represents the capacity of T cells to secrete combinations of 5, 4, 3 or 2 cytokines. The results are shown as the mean ± SEM. *P < 0.05, **P < 0.01 when compared with the HC group

The production of polyfunctional CD8^+^ T cells was high in the LP group under no-stimulation conditions (Fig. 6). Following stimulation with SEB or CL097, the frequencies of CD8^+^ T cells that simultaneously secreted 2, 3, or 4 of the above-referenced cytokines increased in the samples collected from the patients with LP. Although no differences were noted between the groups when the cytokines were combined, a different response was observed upon activation with CL097 (pie chart, Fig. 6).

## Discussion

In this report, we identified systemic immunologic alterations that occur in patients with LP, including altered innate immune responses caused by mDCs secreting TNF and altered effector T cell responses [3]. Moreover, our ex vivo evaluation revealed the presence of polyfunctional CD8^+^ T cells secreting IL-22, IL-17, IFN-γ and/or IL-10 in parallel with increased numbers of Th22 and Tc22 cells and CD8^+^ and CD4^+^Foxp3^+^ Tregs.

Previously, when evaluating PBMCs isolated from patients with LP, we observed an increase in TNF secretion following TLR4 or TLR7/TLR8 activation and a decrease in TNF following TLR7 activation. A G/A polymorphism found at position 308 in the gene encoding TNF is a risk factor for LP, regardless of the presence of HCV infection [21]. Additionally, TNF-α levels are increased in the saliva of individuals with OLP and in the sera of individuals with cutaneous LP, although the function of this cytokine in the context of LP is controversial [3]. Treatment to produce a TNF blockade can lead to the formation of lichenoid reactions in the skin and oral mucosa; as such, further studies are required to better understand the role of this cytokine in LP pathogenesis.

Given the importance of TNF in skin diseases, we evaluated the role of TNF-α in mDC responses to TLR activation using the compound CL097, which binds to both TLR7 and TLR8 (mDCs do not express TLR9). The activation of TLR4 and TLR7/8 induced an increase in TNF responsiveness by mDCs in the LP group. Although differences were not found in the co-stimulatory molecules CD80 and CD86 (data not shown), the high responsiveness of the mDCs to TLR activation could impact T cell responses in patients with LP. To evaluate pDC responses, we used TLR7 and TLR9 agonists because these receptors are selectively expressed by human pDCs [22, 23]. Although these cells are abundant in LP lesions and scarce in blood, we found no differences in the peripheral blood samples collected from the HCs and patients with LP. Previously, we showed that patients with LP have increased serum levels of CXCL9 and CXCL10 [3]. These chemokines share the CXCR3 receptor, which is up-regulated in inflamed tissues as well as in pDCs and mDCs. The activation of these chemokines through this receptor may favour their sequestration in skin lesions. Moreover, we also showed that LP skin lesions exhibit an interferon type I signature that, interestingly, was related to the negative regulation of endogenous retrovirus expression [4] and to pDC infiltration into skin lesions, a characteristic of LP [24]. These cells probably migrate to skin or to secondary lymphoid organs, where they induce adaptive immune responses such as the induction of Tregs.

The balance between regulatory and effector T cells was analysed, and increased percentages of CD4^+^ and CD8^+^ Tregs were detected in the peripheral blood of patients with LP. These peripherally derived Tregs are generated outside of the thymus from CD4^+^CD25^−^ T cell precursors under specific stimulation conditions [25]. Moreover, unlike CD4^+^Foxp3^+^ Tregs, which are generated in the thymus, suppressive CD8^+^Foxp3^+^Tregs appear after primary antigen stimulation, suggesting that these cells are amplified by TCR stimulation, as verified in patients with inflammatory diseases such as autoimmune type 1 diabetes and multiple sclerosis [25, 26]. Although we have not evaluated the function of these cells, the increased frequencies of CD4^+^ and CD8^+^ Tregs in the peripheral blood of patients with LP seem to control inflammatory immune responses in LP skin lesions [27, 28]. Studies of Tregs in the cutaneous form of LP are scarce; the majority of studies have examined OLP.

In PBMCs, the activation of TLRs is primarily thought to affect antigen-presenting cells by inducing an innate immune response that can subsequently activate the adaptive immune system. However, an increasing amount of data has demonstrated that TLRs are expressed and activated in T cells, thus providing evidence for a direct role of TLRs in the activation of the adaptive immune response [29]. In the current study, we found it most appropriate to evaluate the effects of TLR agonists on T cells in vitro while taking into account the involvement of DCs in the immune response to mimic the in vivo environment.

Under no-stimulation conditions, the samples from the LP group exhibited an increased frequency of CD4^+^IL-10^+^ T cells, which decreased upon TLR activation. In addition, TLR4 activation induced the production of CD4^+^IFN-γ^+^ T cells. TLR activation also produced a proinflammatory microenvironment, which was probably modulated by mDCs and may lead to an inflammatory status in patients with LP due to CD4^+^ T cell responses. It is notable that the TLR9 agonist used in this study down-regulated cytokine secretion from both CD4^+^ and CD8^+^ T cells in the LP group. This relationship suggests that this agonist may be an interesting adjuvant to induce tolerance and attenuate inflammatory responses in individuals with LP.

Notably, CD8^+^ T cell activation through TLR7/8 or SEB led to an increased frequency of monofunctional IL-22^+^ T cells. Moreover, increased frequencies of Tc22 and Th22 cells (IFN-γ^−^ and IL-17a^−^) were detected in the patients with LP. These increases could result from the migration of IL-22-secreting T cells to sites of skin/mucosal inflammation. Recent evidence indicates that IL-22 is an important cytokine for the protection and tissue remodelling of the skin, whereas Tc22 cells have been implicated in the pathogeneses of psoriasis and atopic dermatitis [30, 31]. Although the frequencies of the above cell populations were increased in the LP group, the roles of IL-22 and Th22/Tc22 cells in LP are currently not well established and require further study.

Polyfunctional T cells can produce multiple cytokines simultaneously, providing a more effective immune response to a pathogen than cells that produce only a single cytokine [17]. In the LP group, we observed that TLR activation in PBMCs induced the production of polyfunctional T cells, mainly through the activation of the TLR7/TLR8 pathway. Although there were fewer CD4^+^ T cells simultaneously secreting 5 cytokines in the LP group, there was an increased frequency of CD4^+^ T cells simultaneously secreting combinations of 2 or 3 cytokines. Notably, the absence of the cytokine IL-10 seemed to increase polyfunctional CD4^+^ T cell frequency. Multifunctional, Th1-skewed cytokine responses (identified by the simultaneous secretion of IFN-γ, IL-2, and TNF-α) have been described to be initiated asynchronously, although the ensuing dynamic trajectories of these responses evolve in a sequential and systematic manner. In LP, we verified that multifunctional CD4^+^ T cells did not secrete IL-10 upon activation, suggesting a possible defect in the IL-10 pathway in this cell population.

Polyfunctional T cells (i.e., single cells producing two or more immune mediators) play a role in controlling HIV and other persistent infections [18, 32]. Although polyfunctional T cells represent a very low frequency of total CD4^+^ and CD8^+^ T cells (in addition to monofunctional T cells) in HIV patients, these cells could lower viral load and have been associated with long-term suppression of AIDS progression [33]. Moreover, HCV-specific T cell responses have been associated with effective control of HCV replication [34]. Whether levels of unstimulated polyfunctional CD8^+^ T cells increased in association with viral infections in patients with LP must be further explored. In our LP cohort, we observed a high prevalence of previous viral infections (e.g., cytomegalovirus, herpes simplex virus, and Epstein-Barr virus) but no cases of infection with HCV, which is the virus typically associated with LP [4]. Indeed, the aetiology of LP is still unclear, although possible causes include viral infections such as those caused by human herpes virus type 7 or HCV, which induce multifunctional populations of T cells [2, 34].

In summary, we detected a disequilibrium between regulatory and effector functions in T cells in the peripheral blood of patients with LP. Our results indicated that TLR ligands could be used as adjuvants for the modulation of immune responses in LP.

## Conclusions

In the current study, we showed that LP is associated with an altered innate immune response mediated through the responses of TNF-α^+^ mDCs to TLR activation. Alterations in the production of T effector cells with increased Th22/Tc22 subpopulations and impairments in IL-10 secretion from CD4^+^ T cells were induced by TLR stimulation. These results suggest the presence of regulatory cells in the blood of patients with LP, indicating that the inflammatory response associated with LP is not restricted to the skin. Moreover, polyfunctional T cells provide a robust immune response may be useful as an adjuvant for the treatment of LP.

## Additional files

10.1186/s12967-016-0938-1 The gating strategy used to evaluate dendritic cells. The gating strategy used to evaluate mDC (CD11c) and pDC (CD123) populations in PBMCs collected from healthy individuals. The subsequent panel illustrates the population of interest producing each studied cytokine upon SEB (mDCs) and CpG (pDCs) stimulation.
10.1186/s12967-016-0938-1 The gating strategy used to evaluate CD4^+^ Tregs. A representative gating strategy with the selection of CD4^+^ Tregs and subtypes. A similar gating strategy was used for CD8^+^ Treg subsets (data not shown).
10.1186/s12967-016-0938-1 The gating strategy used to evaluate mono- and polyfunctional CD4^+^ T cells. A representative gating strategy used for the selection of CD4^+^ T cells (a). A similar gating strategy was used for CD8^+^ T cell subsets (b). Each subsequent panel depicts only the population of interest producing each studied cytokine after TLR or SEB stimulation. Boolean gating was used to calculate the proportions of polyfunctional T cells (c); a similar gating strategy was used for CD8^+^ polyfunctional T cell subsets.
10.1186/s12967-016-0938-1 Altered cytokine secretion by CD8^+^ T cells upon TLR activation in patients with LP. PBMCs obtained from patients with LP (n = 13, closed circles) and HCs (n = 19, open circles) were left unstimulated (a) or were stimulated with the TLR agonists CL097/TLR7-8, CpG/TLR9, and SEB for 16 h (b) and then assessed for IFN-γ, IL-22 and TNF secretion from CD8^+^ T cells using flow cytometry. The frequencies of stimulated CD3^+^CD8^+^ T cells were subtracted from the unstimulated values. The results are shown as the mean ± SEM. *p < 0.05, **p < 0.01 when compared with the HC group.
10.1186/s12967-016-0938-1 Ratios of Th17 and Th1 cells to Tregs frequencies. PBMCs obtained from patients with LP (n = 6) and HCs (n = 13) were left unstimulated. The results are shown as the mean ± SEM.

## Abbreviations

LP: lichen planus
DC: dendritic cells
TLR: toll-like receptors
pDC: plasmacytoid dendritic cells
mDC: myeloid dendritic cells
IFN-α: interferon alpha
TNF-α: tumour necrosis factor alpha
Tregs: T regulatory cells
IL: interleukin
TNF: tumour necrosis factor
PBMCs: peripheral blood mononuclear cells
tTreg: thymic T regulatory cells
pTreg: peripherally induced T regulatory cells
OLP: oral lichen planus
PMA: phorbol myristate acetate
Th: T helper
LPS: lipopolysaccharide
SEB: enterotoxin B from *Staphylococcus aureus*
HCV: hepatitis C virus
TCR: T cell receptor
AIDS: acquired immunodeficiency syndrome
