# Supplementary figures and images for: Activation of myeloid dendritic cells, effector cells and regulatory T cells in lichen planus
Source: J Transl Med. 2016 Jun 10;14:171. doi: 10.1186/s12967-016-0938-1 (PMC4901415; doi:10.1186/s12967-016-0938-1)

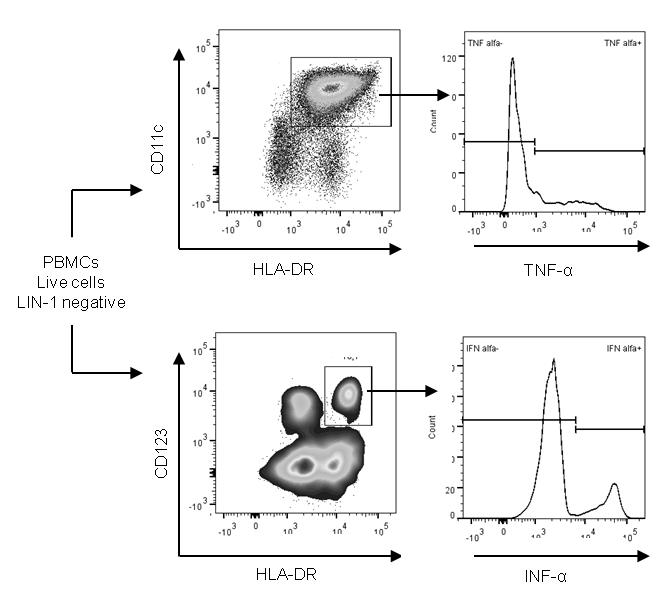

Supplement: Supplementary file 1 — 10.1186/s12967-016-0938-1 The gating strategy used to evaluate dendritic cells. The gating strategy used to evaluate mDC (CD11c) and pDC (CD123) populations in PBMCs collected from healthy individuals. The subsequent panel illustrates the population of interest producing each studied cytokine upon SEB (mDCs) and CpG (pDCs) stimulation. [file 12967_2016_938_MOESM1_ESM.tif]

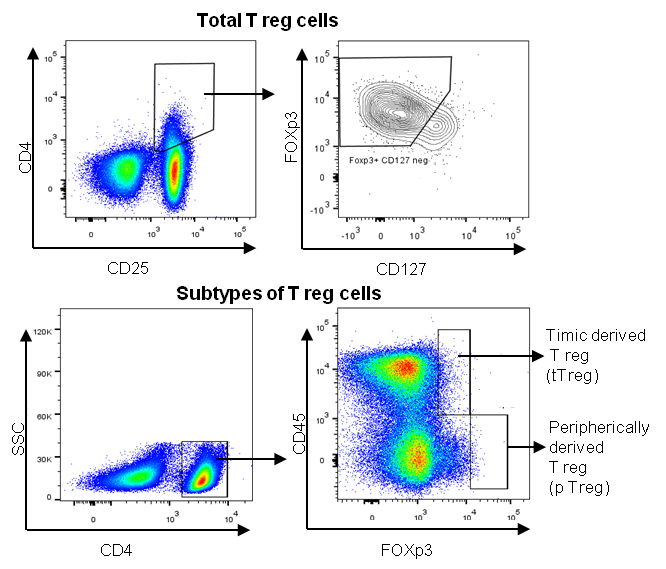

Supplement: Supplementary file 2 — 10.1186/s12967-016-0938-1 The gating strategy used to evaluate CD4+ Tregs. A representative gating strategy with the selection of CD4+ Tregs and subtypes. A similar gating strategy was used for CD8+ Treg subsets (data not shown). [file 12967_2016_938_MOESM2_ESM.tif]

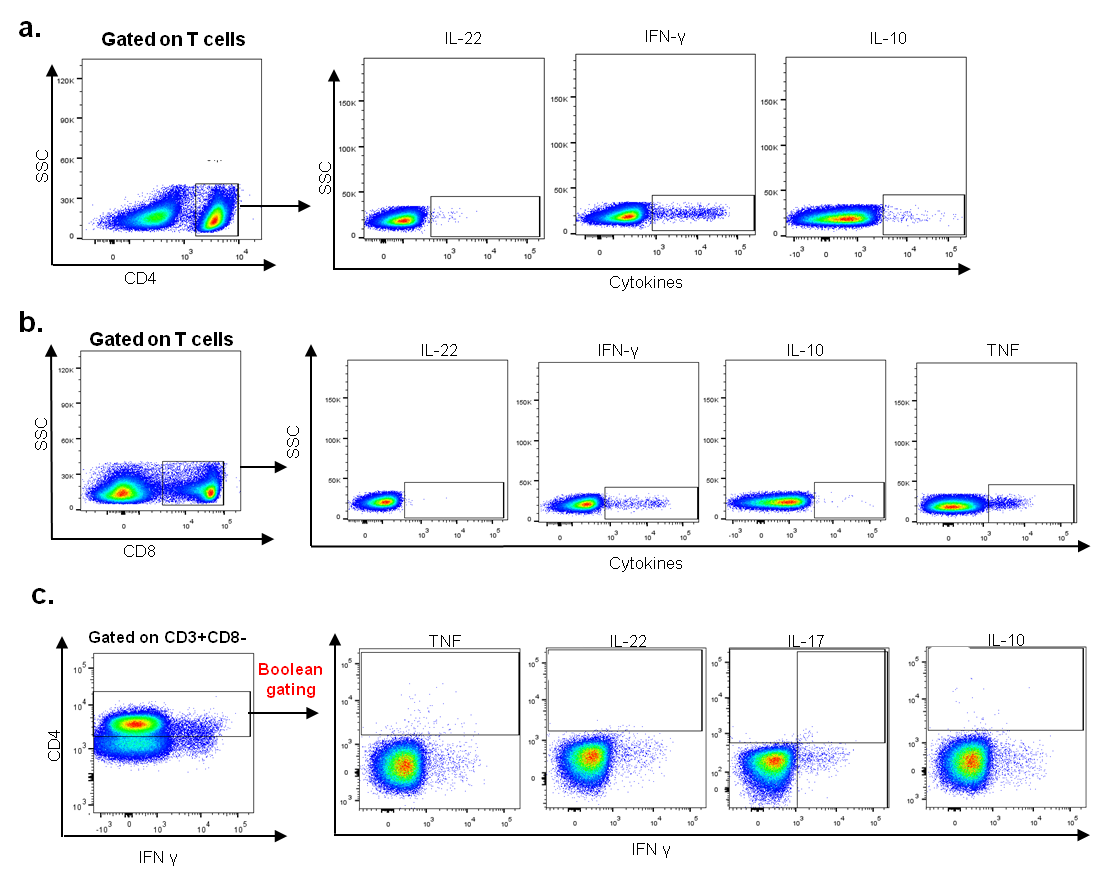

Supplement: Supplementary file 3 — 10.1186/s12967-016-0938-1 The gating strategy used to evaluate mono- and polyfunctional CD4+ T cells. A representative gating strategy used for the selection of CD4+ T cells (a). A similar gating strategy was used for CD8+ T cell subsets (b). Each subsequent panel depicts only the population of interest producing each studied cytokine after TLR or SEB stimulation. Boolean gating was used to calculate the proportions of polyfunctional T cells (c); a similar gating strategy was used for CD8+ polyfunctional T cell subsets. [file 12967_2016_938_MOESM3_ESM.tif]

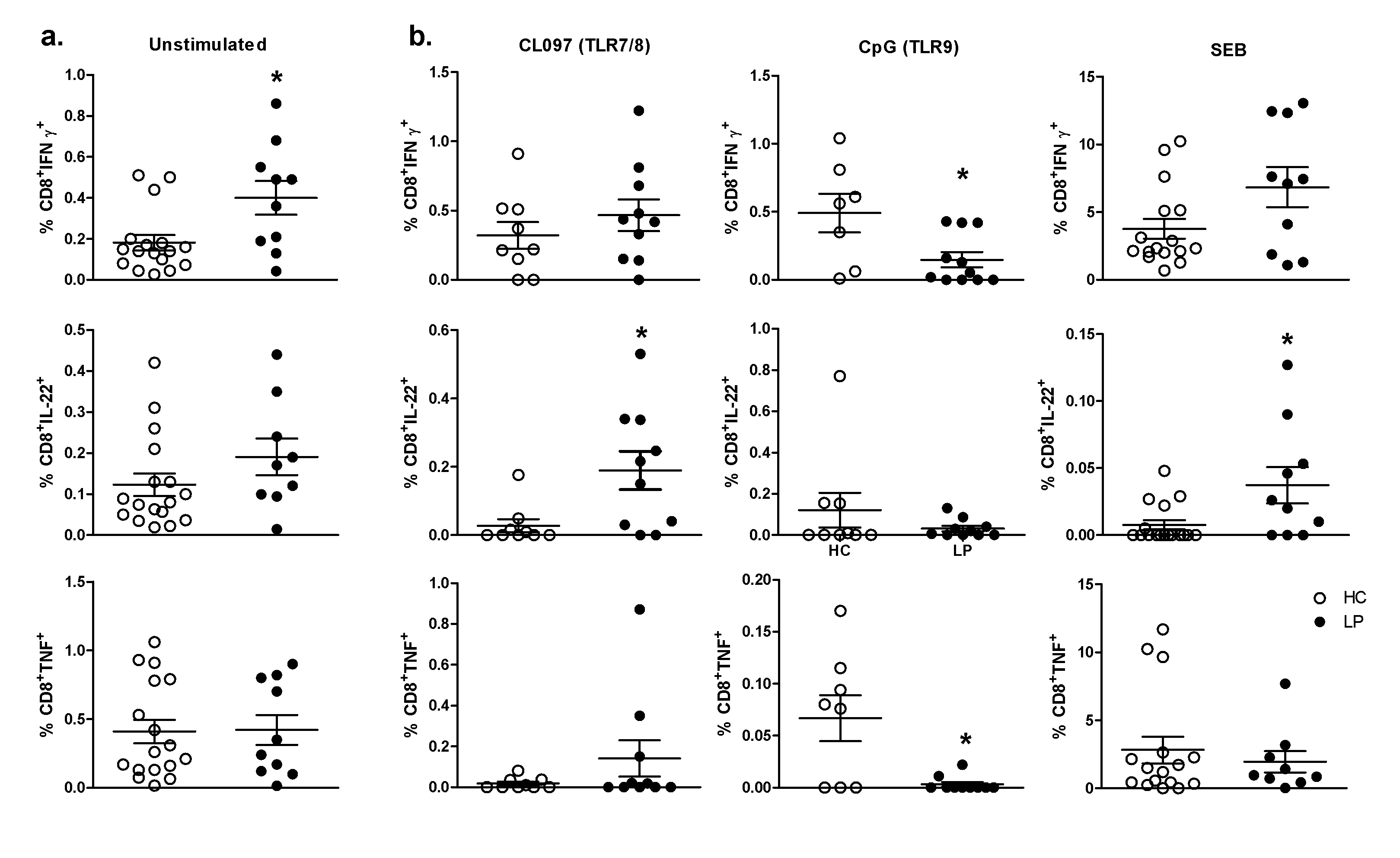

Supplement: Supplementary file 4 — 10.1186/s12967-016-0938-1 Altered cytokine secretion by CD8+ T cells upon TLR activation in patients with LP. PBMCs obtained from patients with LP (n = 13, closed circles) and HCs (n = 19, open circles) were left unstimulated (a) or were stimulated with the TLR agonists CL097/TLR7-8, CpG/TLR9, and SEB for 16 h (b) and then assessed for IFN-γ, IL-22 and TNF secretion from CD8+ T cells using flow cytometry. The frequencies of stimulated CD3+CD8+ T cells were subtracted from the unstimulated values. The results are shown as the mean ± SEM. *p < 0.05, **p < 0.01 when compared with the HC group. [file 12967_2016_938_MOESM4_ESM.tif]

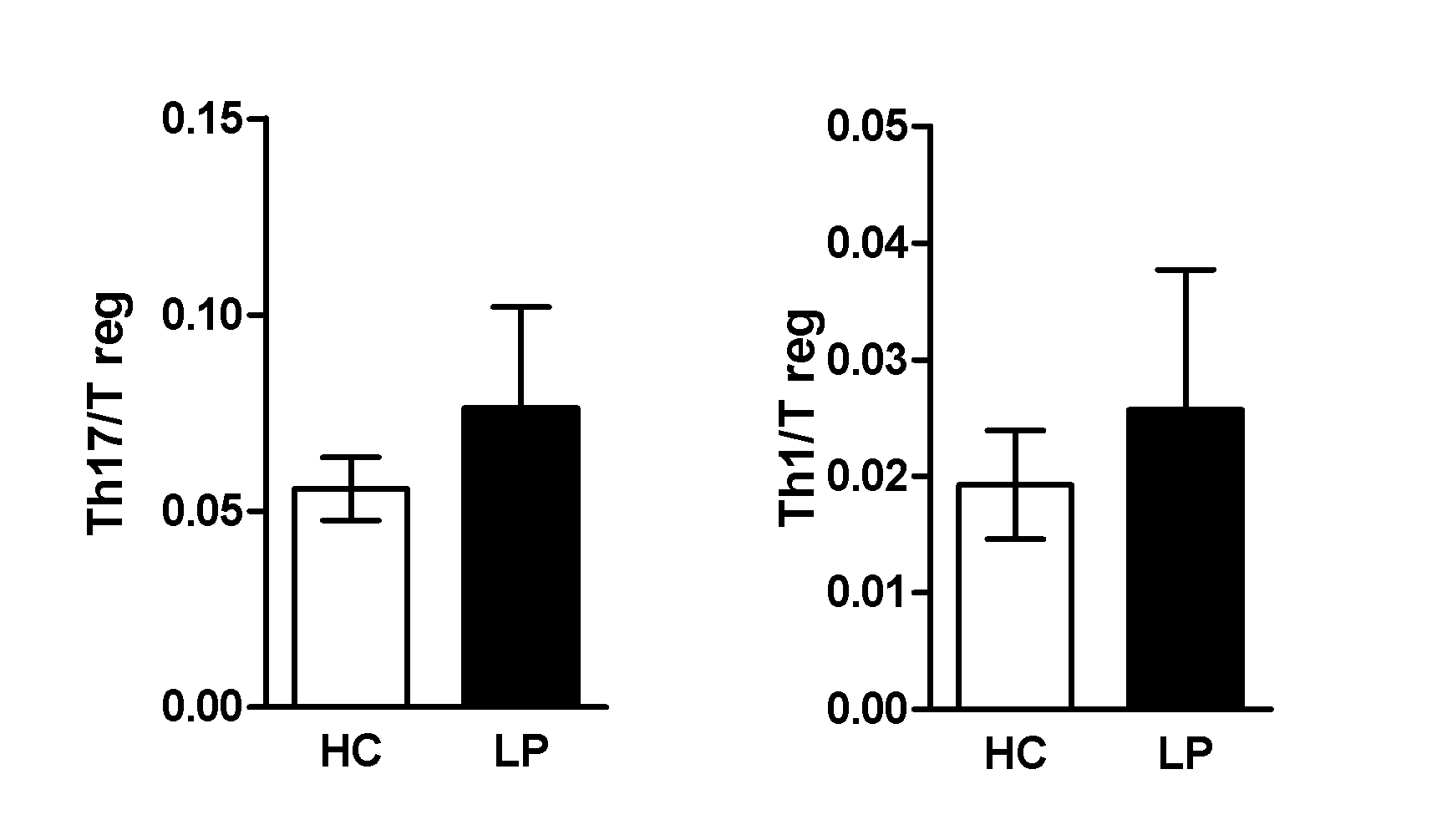

Supplement: Supplementary file 5 — 10.1186/s12967-016-0938-1 Ratios of Th17 and Th1 cells to Tregs frequencies. PBMCs obtained from patients with LP (n = 6) and HCs (n = 13) were left unstimulated. The results are shown as the mean ± SEM. [file 12967_2016_938_MOESM5_ESM.tif]
